# Monocyte-Driven Systemic Biomarkers and Survival After Pulmonary Metastasectomy in Metachronous Lung-Limited Oligometastatic Disease: A Retrospective Single-Center Study

**DOI:** 10.3390/jcm15020476

**Published:** 2026-01-07

**Authors:** Hacer Boztepe Yesilcay, Asim Armagan Aydin, Ahmet Unlu, Sencan Akdag, Kamuran Yuceer, Mustafa Yildiz

**Affiliations:** 1 Department of Thoracic Surgery, University of Health Sciences, Antalya Training and Research Hospital, 07100 Antalya, Turkey; sencanakdag@hotmail.com; 2 Department of Medical Oncology, University of Health Sciences, Antalya Training and Research Hospital, 07100 Antalya, Turkey; drarmaganaydin@gmail.com (A.A.A.); md.ahmetunlu@gmail.com (A.U.); drmyildiz@yahoo.com (M.Y.); 3 Department of Medical Oncology, Erciyes University School of Medicine, 38030 Kayseri, Turkey; drkamuranaltiparmak@gmail.com

**Keywords:** metastasectomy, pulmonary nodule, biomarker, metachronous, lung metastasis, cytoreductive surgery, systemic inflammation response index, monocyte-to-albumin ratio, immune inflammation, prognosis

## Abstract

**Background/Objectives:** Metachronous lung-limited oligometastatic disease represents a biologically heterogeneous state in which patient selection for pulmonary metastasectomy remains challenging. While systemic inflammation–nutrition indices have shown prognostic value across malignancies, their relevance in this strictly defined surgical setting is not well established. **Methods:** We conducted a retrospective single-center cohort study including 109 patients with isolated metachronous pulmonary recurrence who underwent curative intent R0 metastasectomy between September 2015 and April 2024. Preoperative systemic biomarkers, including neutrophil-to-lymphocyte ratio (NLR), systemic immune-inflammation index (SII), systemic inflammation response index (SIRI), pan-immune-inflammation value (PIV), and monocyte-to-albumin ratio (MAR), were evaluated using receiver operating characteristic (ROC) analysis and multivariable Cox models to determine their association with overall survival (OS) and progression-free survival (PFS). Clinicopathological variables, such as lymph node involvement and metastatic burden, were incorporated into the adjusted models. **Results:** The median age of the cohort was 61 years (range, 29–82 years), and the sex distribution was balanced (48.6% female and 51.4% male), with 62.4% of patients being younger than 65 years. Among the systemic indices evaluated, monocyte-weighted biomarkers demonstrated the strongest prognostic performance. The MAR showed the highest discriminative ability for mortality (AUC, 0.749; *p* < 0.001), followed by the SIRI (AUC, 0.682; *p* = 0.007). In multivariable analyses, MAR independently predicted OS (*p* = 0.043) and PFS (*p* = 0.023), while SIRI independently predicted PFS (*p* = 0.043). Lymph node involvement remained the dominant adverse prognostic factor for both outcomes (*p* < 0.001); however, monocyte-weighted indices provided additional prognostic value beyond conventional anatomic criteria. **Conclusions:** Preoperative SIRI and MAR capture host immune–metabolic states that are relevant to postoperative trajectories and may refine risk stratification in candidates for pulmonary metastasectomy. These readily obtainable markers warrant prospective validation within biologically integrated selection frameworks.

## 1. Introduction

Despite substantial advances in curative-intent therapy for early-stage solid tumors, distant relapse continues to occur in 20–40% of patients [1]. Because of its extensive capillary network, high filtration capacity, and permissive microenvironment, the lung is a frequent site of metastatic spread from breast, colorectal, renal, and soft tissue sarcoma primaries [2]. Epidemiological evidence shows that 10–25% of patients in remission eventually develop isolated pulmonary metastases [3], many displaying a limited metastatic burden consistent with the oligometastatic phenotype first proposed by Hellman and Weichselbaum [4]. This concept, characterized by restricted metastatic potential and intermediate biological aggressiveness, has reshaped contemporary views on metastatic disease and provides a rationale for employing potentially curative local therapies in selected patients [5].

Pulmonary metastasectomy has historically been associated with favorable long-term outcomes [6,7,8], with a retrospective series reporting 5-year survival rates of 30–50% in carefully selected patients across multiple tumor types [9,10,11]. These findings likely reflect both the biological selection for indolent metastatic behavior and the technical feasibility of achieving complete cytoreduction. However, the true magnitude of the benefits attributable to surgery remains uncertain [8,12]. The PulMiCC randomized trial, the only prospective evaluation to date, did not demonstrate a clear overall survival advantage, reinforcing concerns about selection bias and the limitations of conventional anatomic criteria such as nodule number, size, disease-free interval, and resectability [13]. Recent analyses highlight the lack of validated tools to guide surgical decision-making and underscore the need for biologically informed prognostic frameworks to refine patient selection [14].

Systemic inflammation and host immunonutritional status have emerged as critical determinants of tumor progression, metastatic competence, and treatment responsiveness [15]. Building on early work with single-parameter markers, such as the neutrophil-to-lymphocyte ratio (NLR) [16], more integrative composite indices, including the systemic immune-inflammation index (SII) [17], systemic inflammation response index (SIRI) [18], pan-immune-inflammation value (PIV) [19], and monocyte-to-albumin ratio (MAR), ref. [20] have been developed to capture the multidimensional interplay among neutrophils, lymphocytes, monocytes, and platelets. These biomarkers mirror broader biological processes linking pro-tumor inflammatory signaling, impaired cell-mediated immunity, and metabolic reserve.

Notably, monocyte-weighted indices may reflect upstream activity within the tumor immune microenvironment, as circulating monocytes serve as precursors for tumor-associated macrophages (TAMs), which are key orchestrators of metastatic niche conditioning, immunosuppressive remodeling, and local treatment resistance [21,22]. Through its influence on angiogenesis, extracellular matrix dynamics, and T-cell exclusion, TAM-driven programs shape both metastatic outgrowth and the host’s capacity to mount an effective antitumor response [23]. Such mechanistic connections provide a biologically coherent rationale for investigating whether immuno-nutrition-based indices carry clinically meaningful information regarding the metastatic behavior of lung-limited oligometastatic disease and a patient’s potential to benefit from metastasis-directed surgery.

Despite the increasing use of these indices across oncology, their prognostic relevance in metachronous, lung-limited oligometastatic disease patients undergoing curative-intent metastasectomy remains poorly defined. Existing data offer limited guidance on whether such biomarkers can meaningfully complement clinicopathological variables or improve postoperative risk assessments. Against this background, the present study systematically evaluates the prognostic performance of five routinely available immune-inflammatory and nutritional indices—NLR, SII, SIRI, PIV, and MAR—in patients undergoing R0 pulmonary metastasectomy for isolated metachronous recurrence. By situating these markers within a biologically coherent framework, we aimed to explore whether they can contribute to a more objective and mechanistically grounded approach to select candidates that are most likely to benefit from metastasis-directed surgery.

## 2. Materials and Methods

### 2.1. Study Design and Setting

**Figure 1:**
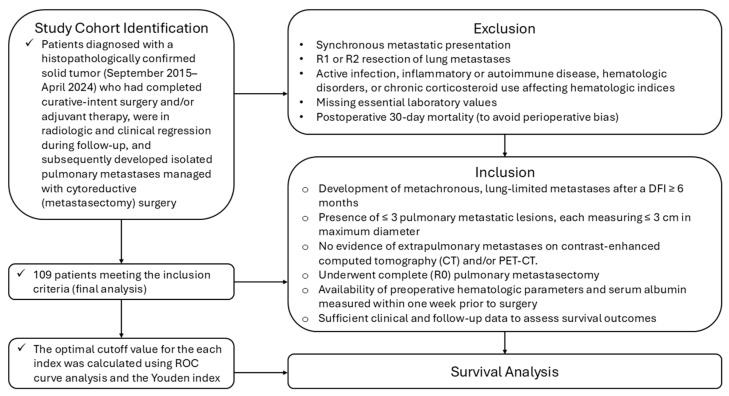
Flow Diagram of Patient Selection, Eligibility Criteria, and Final Study Cohort.

This retrospective single-center cohort study was conducted at the Department of Thoracic Surgery, a high-volume tertiary referral center at the University of Health Sciences Antalya Training and Research Hospital. The study population was identified through a systematic review of all patients evaluated and treated for pulmonary metastases between September 2015 and April 2024; 109 patients met the predefined inclusion and exclusion criteria and were included in the final analysis. Eligible cases were selected based on predefined inclusion and exclusion criteria to establish a biologically and clinically homogeneous cohort (Figure 1). This study specifically investigated the prognostic significance of preoperative systemic inflammatory and nutritional indices in patients with metachronous lung-limited oligometastatic disease who underwent curative-intent pulmonary metastasectomy during the defined study period. None of the patients had received prior systemic chemotherapy or immunotherapy before lung surgery.

### 2.2. Study Population

#### 2.2.1. Definition of Metachronous Lung-Limited Oligometastatic Disease

To ensure biological homogeneity and reduce confounding from active primary disease, only patients with metachronous lung metastases were included in the study. Metachronous metastases were defined as metastatic lesions detected after the completion of definitive treatment for the primary tumor, followed by a disease-free interval (DFI) of at least 6 months. Patients with synchronous disease, defined as lung metastases present at primary diagnosis or developing within 6 months, were excluded because of known biological and clinical heterogeneity (Figure 1).

#### 2.2.2. Inclusion Criteria

Patients were eligible if they met all of the following criteria:

- Histologically confirmed primary solid tumor of any organ origin.
- Development of metachronous, lung-limited metastases after a DFI ≥ 6 months.
- Presence of ≤3 pulmonary metastatic lesions, each measuring ≤3 cm in maximum diameter.
- No evidence of extrapulmonary metastases on Contrast-enhanced computed tomography (CT) and/or positron emission tomography–computed tomography (PET-CT).
- Underwent complete (R0) pulmonary metastasectomy.
- Availability of preoperative hematologic parameters and serum albumin measured within one week prior to surgery.
- Sufficient clinical and follow-up data to assess survival outcomes.

#### 2.2.3. Exclusion Criteria

Patients were excluded if any of the following applied:

- Synchronous metastatic presentation.
- R1 or R2 resection of lung metastases.
- Active infection, inflammatory or autoimmune disease, hematologic disorders, and chronic corticosteroid use affect hematologic indices.
- Missing essential laboratory values.
- Postoperative 30-day mortality (to avoid perioperative bias).

### 2.3. Data Collection

Clinical, radiological, laboratory, and surgical data were retrospectively retrieved from the institutional electronic medical record system. The recorded baseline variables included age, sex, smoking status, comorbidities, and Eastern Cooperative Oncology Group (ECOG) performance status. Primary tumor characteristics, tumor site, histologic subtype, prior curative-intent therapy (surgery, adjuvant chemotherapy, and/or adjuvant radiotherapy), and disease-free intervals were documented for all patients. Pulmonary metastatic disease was characterized by the number and size of metastatic lesions, laterality, lobe-specific anatomic distribution, and presence of intrathoracic lymph node involvement on contrast-enhanced CT or PET-CT. Operative data included the type of pulmonary resection (wedge resection, segmentectomy, or lobectomy), surgical approach (VATS or open thoracotomy), mediastinal lymph node sampling, and final margin status. Postoperative information included perioperative complications, administration of postoperative therapies, and findings of routine surveillance imaging. Longitudinal follow-up included documentation of recurrence, recurrence site and timing, survival status, and the date of last contact. All hematological and biochemical values used to calculate systemic inflammatory and nutritional indices were obtained from blood tests performed within 7 days prior to pulmonary metastasectomy.

### 2.4. Definition and Calculation of Hematologic Indices

Preoperative systemic inflammatory and nutritional indices were calculated using standard formulas. The neutrophil-to-lymphocyte ratio (NLR) was defined as the absolute neutrophil count divided by the lymphocyte count [16]; the systemic immune-inflammation index (SII) as platelet count multiplied by neutrophil count divided by lymphocyte count [17]; the systemic inflammation response index (SIRI) [18] as monocyte count multiplied by neutrophil count and divided by lymphocyte count; the pan-immune-inflammation value (PIV) [19] as platelet, neutrophil, and monocyte counts multiplied together and divided by lymphocyte count; and the monocyte-to-albumin ratio (MAR) [20] as the monocyte count divided by the serum albumin concentration. Optimal prognostic cutoff values for each biomarker were determined using receiver operating characteristic (ROC) curve analysis with the Youden index, based on overall survival (OS), and patients were subsequently classified into high or low biomarker groups accordingly.

### 2.5. Surgical Procedure

All pulmonary metastasectomy procedures were performed with a curative intent by experienced thoracic surgeons at our institution. The surgical approach and extent of resection were individualized based on the size, number, and anatomical location of the metastatic lesions, with priority given to achieving complete tumor clearance while preserving the lung parenchyma. Surgical techniques included wedge resection, anatomical segmentectomy, or lobectomy, which were selected according to lesion accessibility and oncologic appropriateness. Video-assisted thoracoscopic surgery (VATS) is preferred when feasible, whereas open thoracotomy is performed in patients requiring complex resections or when minimally invasive access is not suitable. Complete resection (R0) was confirmed by histopathological examination of the surgical specimens. Mediastinal lymph node sampling or dissection was performed at the discretion of the operating surgeon, particularly in cases of radiological suspicion of lymphadenopathy or central lesion location.

### 2.6. Follow-Up and Outcome Assessment

Postoperative surveillance was conducted according to institutional thoracic oncology protocols, and included contrast-enhanced chest CT imaging every three months during the first two years and every six months thereafter. At each follow-up visit, patients underwent clinical evaluation, symptom assessment, and interval imaging to document the disease status and any new treatments or complications. Disease recurrence was defined as the presence of new metastatic lesions within the lung, mediastinum, or distant organs. The date and anatomical site of recurrence were recorded for all patients. Time-to-event outcomes were assessed using standardized definitions. OS was defined as the interval from pulmonary metastasectomy to death from any cause or the last confirmed follow-up, with surviving patients censored at their most recent clinical contact. Progression-free survival (PFS) was defined as the time from metastasectomy to the first documented recurrence of intrapulmonary, regional, distant, or death from any cause, whichever occurred first. Patients without recurrence or death were censored at the date of their last radiological evaluation. These outcome definitions provide a comprehensive assessment of both long-term survival and disease control following curative-intent pulmonary metastasectomy.

### 2.7. Statistical Analysis

All statistical analyses were performed using SPSS Statistics (version 27.0; IBM Corp., Armonk, NY, USA). Continuous variables were summarized as means with standard deviations or medians with interquartile ranges, based on distributional testing using the Shapiro–Wilk method, and were compared using Student’s *t*-test or Mann–Whitney U test, as appropriate. Categorical variables are presented as counts and percentages and were compared using the χ^2^ test or Fisher’s exact test. OS and PFS were analyzed using the Kaplan–Meier method, and between-group differences were assessed using the log-rank test. Univariate Cox proportional hazards models were used to identify the variables associated with OS and PFS. Factors with a *p*-value < 0.10 in univariable analyses were entered into multivariable Cox regression to determine independent prognostic indicators. The proportional hazard assumption was evaluated using log-minus-log survival plots. The discriminatory performance of systemic inflammatory and nutritional indices was assessed using ROC curve analysis, and areas under the curve (AUCs) were calculated to estimate prognostic accuracy. The optimal cutoff values for each biomarker were determined using the Youden index. All statistical tests were two-sided, and statistical significance was set at *p* < 0.05.

## 3. Results

### 3.1. Baseline Characteristics

The median age of the study cohort was 61 years (range, 29–82 years). A total of 62.4% of the patients were younger than 65 years, and the sex distribution was balanced (48.6% female; 51.4% male). Non-smokers comprised 62.4% of the population and 49.5% had no documented comorbidities. ECOG performance status was 0–1 in 56.9% of patients, whereas 43.1% presented with an ECOG score of ≥2. The gastrointestinal tract was the most frequent primary tumor origin (51.4%), followed by breast (18.3%), gynecological (11.9%), and genitourinary malignancies (10.1%). Metastatic pulmonary lesions were most commonly located in the left upper lobe (29.4%) and the majority were ≥1 cm in diameter (82.6%). Video-assisted thoracoscopic surgery (VATS) was the predominant surgical approach (83.5%), with wedge resection accounting for 78% of the procedures. A prior history of adjuvant chemotherapy and radiotherapy was reported in 78.9% and 36.7% of the patients, respectively. Synchronous lymph node involvement was present in 25.7% of the patients. Lung metastases were categorized as solid (66.1%) or multiple (33.9%). The postoperative complication rate was 6.4% and the median length of hospital stay was 3 days (range, 1–11 days). Comprehensive clinicopathological characteristics of the entire cohort, stratified according to the SIRI and MAR cut-off values, are summarized in Table 1.

When stratified by SIRI, most patients were classified into the low SIRI group (<2.5) (79.8%). Demographic and clinical variables included age, sex, surgical approach, type of resection, Eastern Cooperative Oncology Group (ECOG) performance status, smoking history, comorbidities, prior cancer treatments, length of hospital stay, primary tumor origin, lymph node involvement, and metastatic pattern (all *p* > 0.05). Tumor size >1 cm demonstrated only a borderline higher frequency in the high SIRI group (≥2.5) (*p* = 0.062). In contrast, inflammation-based indices exhibited marked differences; elevated NLR (≥4.5), SII (≥446.5), and PIV (≥209.5) were significantly more common among patients with high SIRI (all *p* < 0.01) (Table 1).

For MAR, patients were evenly distributed across low (<1.46, 49.5%) and high (≥1.46, 50.5%) categories. Higher MAR levels were associated with older age (≥65 years) (46.3% vs. 29.1%, *p* = 0.046) and male sex (63.0% vs. 40.0%, *p* = 0.013). Other demographic and clinical variables, including surgical modality, resection type, ECOG performance status, smoking status, comorbidity burden, tumor origin, lesion characteristics, lymph node involvement, and postoperative complications, did not differ significantly between the MAR groups (all *p* > 0.05). Although a tumor diameter ≥1 cm was more frequent in the high-MAR group, the difference was not significant (*p* = 0.070). MAR levels were not significantly associated with other systemic inflammatory indices (NLR, SII, PIV, and SIRI) (all *p* > 0.05) (Table 1).

### 3.2. ROC-Based Predictive Accuracy of Systemic Inflammation–Nutrition Indices

**Figure 2:**
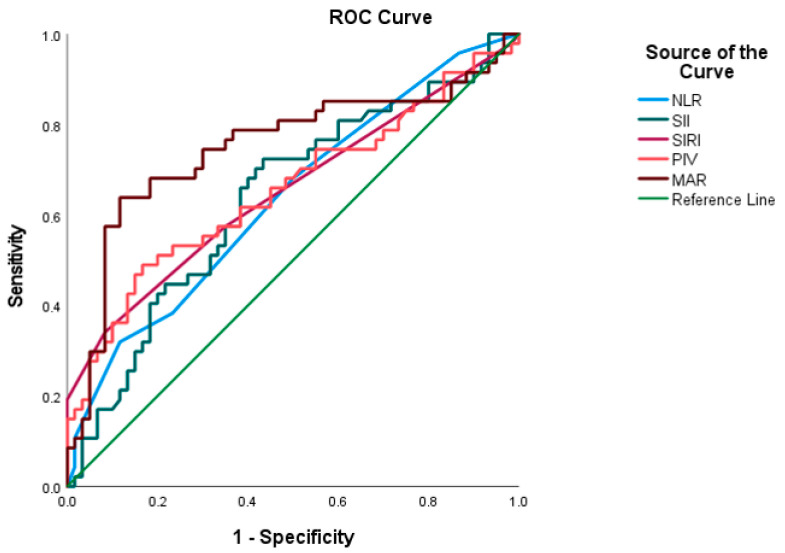
ROC Curves Demonstrating the Predictive Performance of Preoperative Systemic Inflammation–Nutrition Indices for Overall Mortality.

ROC analysis demonstrated variable discriminatory performance among the evaluated systemic inflammation–nutrition indices for predicting the overall mortality. Among all the markers, MAR exhibited the highest prognostic accuracy, with an AUC of 0.749 (95% CI, 0.647–0.850; *p* < 0.001). Using a cut-off value of 1.46, MAR yielded a sensitivity of 74.5% and specificity of 68.3%, indicating clinically meaningful discriminatory capacity. SIRI showed the second highest predictive performance (AUC: 0.682; 95% CI: 0.545–0.760; *p* = 0.007), characterized by high sensitivity (85.1%) and moderate specificity (72.7%). PIV (AUC: 0.655; 95% CI, 0.547–0.763; *p* = 0.006) and SII (AUC: 0.637; 95% CI, 0.530–0.744; *p* = 0.015) demonstrated comparable but modest discriminatory abilities, with PIV providing higher specificity (76.7%). NLR showed the lowest predictive capacity among the tested indices (AUC: 0.635; 95% CI: 0.529–0.741; *p* = 0.017), with limited discriminative value relative to the other markers (Table 2) (Figure 2).

### 3.3. Survival Analysis

During a median follow-up of 31 months, disease progression occurred in 62 patients (56.9%) and 49 patients (45.0%) died. The median PFS for the entire cohort was 32 months (95% CI, 15.2–48.8), and the median OS was 48 months (95% CI, 42.6–53.5).

**Figure 3:**
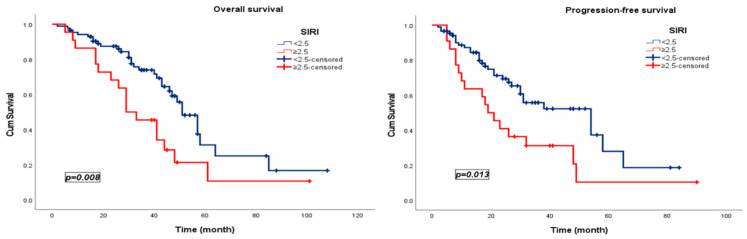
Kaplan–Meier survival curves stratified by the SIRI in patients treated with pulmonary metastasectomy.

Patients in the low-SIRI group (<2.5) experienced substantially improved survival compared to those in the SIRI (≥2.5). The median PFS was more than double in the low-SIRI cohort (51 months; 95% CI, 44.0–57.9) relative to the high-SIRI group (19 months; 95% CI, 12.1–25.9; *p* = 0.013). A similar gradient was observed for OS, with patients with low SIRI (<2.5) achieving a median OS of 54 months (95% CI, 28.8–79.2) compared with 29 months (95% CI, 18.4–39.6) among those with high SIRI (≥2.5) (*p* = 0.008) (Figure 3).

**Figure 4:**
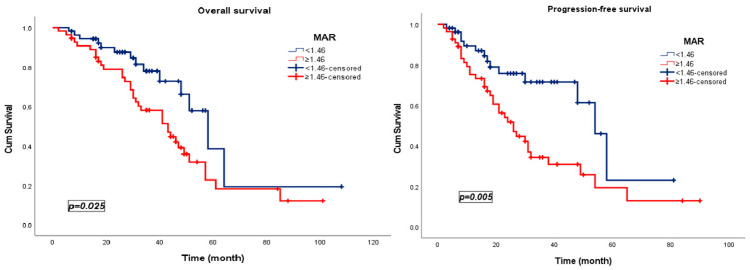
Kaplan–Meier survival curves stratified by the MAR in patients treated with pulmonary metastasectomy.

Patients with low MAR levels (<1.46) demonstrated significantly superior survival outcomes compared with those in the high-MAR group (≥1.46). Specifically, low-MAR patients exhibited a markedly prolonged median PFS of 54 months (95% CI, 45.9–62.1) versus 26 months (95% CI, 18.6–33.4) among high-MAR patients (*p* = 0.005). A parallel pattern was observed for OS, with median OS reaching 58 months (95% CI, 45.6–70.5) in the low-MAR cohort compared with 43 months (95% CI, 31.3–54.6) in those with high MAR (≥1.46) (*p* = 0.025) (Figure 4).

**Figure 5:**
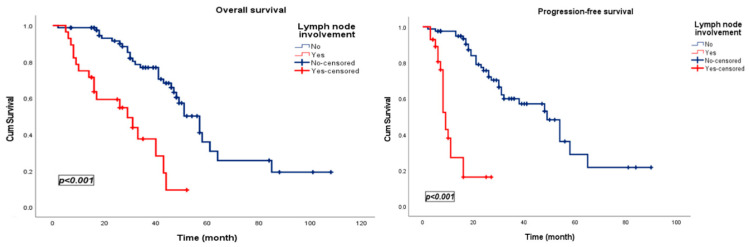
Kaplan–Meier survival curves stratified by the lymph node involvement in patients treated with pulmonary metastasectomy.

Nodal disease was associated with significantly worse outcomes, with markedly reduced OS (29 vs. 57 months, *p* < 0.001) and PFS (9 vs. 49 months, *p* < 0.001) (Figure 5).

### 3.4. Univariate and Multivariate Cox Models Evaluating Prognostic Determinants of Survival Outcomes

In univariate analyses, lymph node involvement emerged as the strongest predictor of OS (HR, 4.438; 95% CI, 2.360–8.344; *p* < 0.001). Elevated SIRI levels were also significantly associated with increased mortality risk (HR: 2.180; *p* = 0.010), as was a high MAR score (HR: 2.128; *p* = 0.021, respectively). Conversely, age, sex, type of resection, tumor size, and other inflammation-based indices, including NLR, SII, and PIV, were not demonstrate a statistically significantly associated with OS (Table 3). Similarly, lymph node involvement showed a robust adverse prognostic effect on PFS (HR: 10.101; 95% CI, 4.975–20.510; *p* < 0.001). A higher number of metastatic pulmonary nodules was associated with an increased risk of progression (HR, 2.001; *p* = 0.017). Among the hematologic indices, elevated SIRI (HR: 2.067; *p* = 0.016) and MAR (HR: 2.445; *p* = 0.006) were significant predictors of shorter PFS. In contrast, NLR, SII, PIV, tumor size, and other clinical variables showed no significant association with PFS (Table 3).

In the multivariate Cox regression model, lymph node involvement was the strongest independent predictor of OS (HR, 4.526; 95% CI, 2.340–8.751; *p* < 0.001). In addition, elevated MAR levels independently predicted poor OS (HR: 2.002; *p* = 0.043) (Table 4). For PFS, lymph node involvement demonstrated a profound adverse prognostic effect (HR, 14.124; 95% CI, 6.089–32.761; *p* < 0.001). A higher number of metastatic pulmonary nodules was also independently associated with shorter PFS (HR: 2.019; *p* = 0.024). Among the systemic inflammation–nutrition indices, both elevated SIRI (HR: 1.893; *p* = 0.043) and MAR (HR: 2.189; *p* = 0.023) were independent predictors of progression (Table 4).

## 4. Discussion

In this stringently defined cohort of patients with metachronous, lung-limited oligometastatic disease undergoing curative-intent pulmonary metastasectomy, monocyte-related systemic inflammation–nutrition indices, particularly the SIRI and MAR, emerged as informative predictors of postoperative outcomes. Both markers retained independent associations with overall and progression-free survival, even after accounting for well-established prognostic determinants, such as lymph node involvement and metastatic burden. These findings suggest that composite indices integrating monocyte-driven inflammation and nutritional reserves may capture host-related biological dimensions not reflected in conventional anatomic criteria, offering potential value in refining risk stratification within a population where outcomes remain heterogeneous despite careful surgical selection.

The biological plausibility of these observations is supported by the central role of circulating monocytes and their differentiation into tumor-associated macrophages (TAMs), which orchestrate multiple dimensions of metastatic progression [23,24]. TAMs not only facilitate extracellular matrix remodeling and support angiogenesis but also actively sculpt an immunosuppressive microenvironment through modulation of cytokine signaling, antigen presentation, and T-cell exhaustion pathways. Increasing evidence indicates that monocyte/TAM-driven mechanisms contribute to the establishment of a pre-metastatic niche characterized by stromal activation, recruitment of myeloid-derived suppressor cells, and reprogramming of resident immune populations, which collectively enhance metastatic fitness and impair antitumor surveillance [25]. The functional polarization of macrophages toward protumorigenic M2-like phenotypes has been directly associated with early recurrence and inferior survival across multiple solid tumors, underscoring the relevance of monocyte-weighted systemic indices as potential surrogates of this biology [26]. Albumin, incorporated into the MAR metric, reflects both nutritional integrity and chronic inflammatory burden, which are two host-related factors linked to reduced treatment tolerance, compromised innate and adaptive immunity, and delayed recovery after major surgery [27]. Together, these mechanistic considerations support the interpretation that elevated monocyte-weighted biomarkers reflect a broader unfavorable immune–metabolic milieu that may predispose patients to early postoperative relapse despite technically successful metastasectomy.

Systemic inflammatory markers have been studied in the context of pulmonary metastasectomy for more than a decade, and elevated NLR, CRP, fibrinogen, and other acute-phase reactants have been repeatedly associated with adverse outcomes in heterogeneous surgical cohorts [28,29,30]. However, such markers primarily reflect neutrophil-dominant or hepatic acute-phase responses, which are highly sensitive to transient physiological stimuli and lack specificity for tumor-driven inflammation. Prior studies also frequently combined synchronous and metachronous metastases, including multiorgan metastatic disease, or enrolled patients in close proximity to systemic chemotherapy or immunotherapy, which substantially confounded the biological interpretation of circulating inflammatory markers [29,30]. These methodological limitations have led to inconsistent conclusions and impeded the integration of such biomarkers into clinical decision-making. By focusing exclusively on a well-defined population with isolated metachronous pulmonary recurrence and by evaluating monocyte-weighted indices that incorporate both inflammatory activation and nutritional reserve, our study provides a more biologically coherent framework. This design reduces the variability arising from treatment-related inflammation, systemic disease burden, and temporal heterogeneity, thereby enabling a clearer assessment of how host-related systemic biology contributes to survival after metastasis-directed therapy.

From a clinical standpoint, the persistence of SIRI and MAR as independent prognostic markers, even alongside dominant anatomic determinants, such as nodal involvement and metastatic burden, highlights the complementary nature of systemic host biology in shaping outcomes after pulmonary metastasectomy. Although such biomarkers should not be used in isolation to deny potentially beneficial surgery, they may help identify individuals at a heightened risk of early recurrence despite technically complete resection. Conversely, low biomarker levels, particularly in the context of single-nodule disease and the absence of nodal involvement, may help define a biologically favorable subgroup that is most likely to derive durable benefits. Integrating systemic inflammation–nutrition indices into multidisciplinary deliberations could therefore enhance the objectivity and biological grounding of existing selection algorithms, guide patient counseling, and inform postoperative surveillance intensity.

The credibility of our findings is reinforced by several methodological strengths. The cohort was defined using stringent eligibility criteria to enrich for a biologically coherent group with metachronous, lung-limited oligometastatic disease and no recent systemic therapy exposure. All patients underwent R0 resection in a high-volume thoracic oncology program with standardized perioperative management, thereby reducing variability in surgical technique and follow-up. Parallel evaluation of multiple systemic inflammation–nutrition indices using a consistent methodology allowed direct comparison of their prognostic utility and highlighted the relative superiority of monocyte-weighted markers. These features collectively enhance the internal validity of the study and support its contribution in areas where high-quality prognostic data remain limited.

The limitations of this study warrant further consideration. As this was a retrospective, single-center analysis with a relatively modest sample size, unmeasured confounding factors and selection bias cannot be excluded. The relatively high proportion of patients with an ECOG performance status ≥2 should also be acknowledged, as this likely reflects real-world referral patterns in patients with metachronous metastatic recurrence undergoing pulmonary metastasectomy; while this may introduce additional clinical heterogeneity, it simultaneously enhances the external validity of the findings. Although restricting the cohort to metachronous, lung-limited recurrence improved biological homogeneity, the inclusion of diverse primary tumor types may still introduce residual heterogeneity. Biomarker measurements obtained at a single time point do not capture longitudinal fluctuations in the systemic inflammation or nutritional status. Additionally, the lack of genomic or immunophenotypic profiling limits mechanistic inference regarding the pathways linking monocyte-driven systemic biology to metastatic behavior. External validation in independent cohorts and prospective studies incorporating tissue-based immune analyses are essential to confirm these findings and clarify their potential role in metastasectomy selection algorithms.

## 5. Conclusions

In conclusion, monocyte-weighted systemic inflammation–nutrition indices, particularly SIRI and MAR, offer independent prognostic information in patients undergoing curative-intent pulmonary metastasectomy for metachronous lung-limited oligometastatic disease. These readily available biomarkers appear to capture the host immune–metabolic reserve beyond conventional anatomic criteria and may help refine preoperative risk stratification. Validation in prospective, biologically integrated cohorts is needed to determine their role within future patient selection frameworks for metastasis-directed surgery.

## Figures and Tables

**Table 1:** Clinicodemographic Characteristics by SIRI and MAR Stratification (*n* = 109).

Variable, *n* (%)			SIRI, *n* (%)			MAR, *n* (%)		
			Low (<2.5) ^†^	High (≥2.5) ^†^	*p*	Low (<1.46) ^†^	High (≥1.46) ^†^	*p*
Age	<65	68 (62.4)	56 (64.4)	12 (54.5)	0.271	39 (70.9)	29 (53.7)	0.046
	≥65	41 (37.6)	31 (35.6)	10 (45.5)		16 (29.1)	25 (46.3)	
Sex	Female	53 (48.6)	43 (49.4)	10 (45.5)	0.463	33 (60.0)	20 (37.0)	0.013
	Male	56 (51.4)	44 (50.6)	12 (54.5)		22 (40.0)	34 (63.0)	
Smoking	No	68 (62.4)	53 (60.9)	15 (68.2)	0.356	31 (56.4)	37 (68.5)	0.133
	Yes	41 (37.6)	34 (39.1)	7 (31.8)		24 (43.6)	17 (31.5)	
Comorbidity	No	54 (49.5)	44 (50.6)	10 (45.5)	0.425	31 (56.4)	23 (42.6)	0.106
	Yes	55 (50.5)	43 (49.4)	12 (54.5)		24 (43.6)	31 (57.4)	
ECOG PS	0–1	62 (56.9)	52 (59.8)	10 (45.5)	0.166	32 (58.2)	30 (55.6)	0.467
	≥2	47 (43.1)	35 (40.2)	12 (54.5)		23 (41.8)	24 (44.4)	
Primary tumor origin	Breast	20 (18.3)	18 (20.7)	2 (9.1)	0.439	11 (20.0)	9 (16.7)	0.487
	Gastrointestinal	56 (51.4)	44 (50.6)	12 (54.5)		27 (49.1)	29 (53.7)	
	Gynecologic	13 (11.9)	9 (10.3)	4 (18.2)		10 (18.2)	3 (5.6)	
	Genitourinary	11 (10.1)	8 (9.2)	3 (13.6)		3 (5.5)	8 (14.8)	
	Others	9 (8.3)	8 (9.2)	1 (4.5)		4 (7.3)	5 (9.3)	
Tumor localization	Left upper	32 (29.4)	22 (25.3)	10 (45.5)	0.894	16 (29.1)	16 (29.6)	0.665
	Right upper	28 (25.7)	26 (29.9)	2 (9.1)		13 (23.6)	15 (27.8)	
	Left lower	23 (21.1)	20 (23.0)	3 (13.6)		12 (21.8)	11 (20.4)	
	Right lower	21 (19.3)	16 (18.4)	5 (22.7)		11 (20.0)	10 (18.5)	
	Middle	5 (4.6)	3 (3.4)	2 (9.1)		3 (5.5)	2 (3.7)	
Tumor size	<1 cm	19 (17.4)	18 (20.7)	1 (4.5)	0.062	13 (23.6)	6 (11.1)	0.070
	≥1 cm	90 (82.6)	69 (79.3)	21 (95.5)		42 (76.4)	48 (88.9)	
Surgical procedure	VATS	91 (83.5)	72 (82.8)	19 (86.4)	0.484	46 (83.6)	45 (83.3)	0.186
	Thoracotomy	18 (16.5)	15 (17.2)	3 (13.6)		9 (16.4)	9 (16.7)	
Resection type	Wedge	85 (78.0)	66 (78.6)	19 (86.4)	0.313	41 (74.5)	44 (86.3)	0.102
	Segmentectomy /Lobectomy	24 (22.0)	18 (21.4)	3 (13.6)		14 (25.5)	7 (13.7)	
Adjuvant chemo	No	23 (21.1)	18 (20.7)	5 (22.7)	0.519	9 (16.4)	14 (25.9)	0.161
	Yes	86 (78.9)	69 (79.3)	17 (77.3)		46 (83.6)	40 (74.1)	
Adjuvant RT	No	69 (63.3)	56 (64.4)	13 (59.1)	0.412	34 (61.8)	35 (64.8)	0.450
	Yes	40 (36.7)	31 (35.6)	9 (40.9)		21 (38.2)	19 (35.2)	
Lymph node involvement	No	81 (74.3)	66 (75.9)	15 (68.2)	0.314	41 (74.5)	40 (74.1)	0.564
	Yes	28 (25.7)	21 (24.1)	7 (31.8)		14 (25.5)	14 (25.9)	
Number of pulmonary metastases	Solitary	72 (66.1)	59 (67.8)	13 (59.1)	0.298	40 (72.7)	32 (59.3)	0.100
	Multiple	37 (33.9)	28 (32.2)	9 (40.9)		15 (27.3)	22 (40.7)	
Postoperative complications	No	102 (93.6)	82 (94.3)	20 (90.9)	0.431	53 (96.4)	49 (90.7)	0.211
	Yes	7 (6.4)	5 (5.7)	2 (9.1)		2 (3.6)	5 (9.3)	
NLR, (4.5) ^†^	Low	22 (20.2)	8 (9.2)	14 (63.6)	<0.001	10 (18.2)	12 (22.2)	0.387
	High	87 (79.8)	79 (90.8)	8 (36.4)		45 (81.8)	42 (77.8)	
SII, (446.5) ^†^	Low	25 (22.9)	25 (28.7)	0 (0.0)	0.002	13 (23.6)	12 (22.2)	0.521
	High	84 (77.1)	62 (71.3)	22 (100.0)		42 (76.4)	42 (77.8)	
PIV, (209.5) ^†^	Low	21 (19.3)	21 (24.1)	0 (0.0)	0.005	13 (23.6)	8 (14.8)	0.178
	High	88 (70.7)	66 (75.9)	22 (100.0)		42 (76.4)	46 (85.2)	

Abbreviations: ECOG PS, Eastern Cooperative Oncology Group performance status; VATS, Video-Assisted Thoracoscopic Surgery; RT, Radiotherapy; NLR, Neutrophil-to-lymphocyte ratio; SII, Systemic immune-inflammation index; SIRI, Systemic inflammation response index; PIV, Pan-immune-inflammation value; MAR, Monocyte-to-albumin ratio; ^†^, predefined cut-off value established for each biomarker.

**Table 2:** Predictive Accuracy of Preoperative Systemic Inflammation–Nutrition Indices for Overall Mortality According to ROC Analysis.

Marker	AUC	Std. Error	*p*	95% CI		Sensitivity (%)	Specificity (%)	Cut-Off
				Lower	Upper			
NLR	0.635	0.054	0.017	0.529	0.741	69.1	88.3	4.5
SII	0.637	0.054	0.015	0.530	0.744	66.0	91.7	446.5
SIRI	0.682	0.055	0.007	0.545	0.760	85.1	72.7	2.5
PIV	0.655	0.055	0.006	0.547	0.763	85.1	76.7	209.5
MAR	0.749	0.052	<0.001	0.647	0.850	74.5	68.3	1.46

Abbreviations: AUC, Area under the curve; Std., standard deviation; CI, Confidence interval; NLR, Neutrophil-to-lymphocyte ratio; SII, Systemic immune-inflammation index; SIRI, Systemic inflammation response index; PIV, Pan-immune-inflammation value; MAR, Monocyte-to-albumin ratio.

**Table 3:** Univariate Cox Regression Analysis of Clinicopathological Variables and Systemic Immune–Nutritional Indices for Overall and Progression-Free Survival.

Overall Survival	Exp(B)	95.0% CI for Exp(B)		*p*	Progression-Free Survival	Exp(B)	95.0% CI for Exp(B)		*p*
		Lower	Upper				Lower	Upper	
Age	1.085	0.611	1.924	0.781	Age	1.082	0.606	1.930	0.790
Sex	0.878	0.487	1.581	0.664	Sex	0.980	0.552	1.742	0.946
Surgical procedure	0.579	0.259	1.294	0.183	Surgical procedure	0.570	0.255	1.273	0.170
Type of resection	0.433	0.170	1.100	0.078	Type of resection	0.417	0.164	1.058	0.066
ECOG PS	1.136	0.646	1.998	0.657	ECOG PS	1.105	0.629	1.942	0.729
Smoking status	1.290	0.725	2.295	0.387	Smoking status	1.169	0.660	2.072	0.592
Comorbidity	1.682	0.940	3.008	0.080	Comorbidity	1.543	0.859	2.773	0.146
Adjuvant chemo	1.106	0.516	2.368	0.796	Adjuvant chemo	1.293	0.604	2.769	0.508
Adjuvant RT	1.429	0.805	2.535	0.223	Adjuvant RT	1.501	0.847	2.660	0.164
Primary tumor origin	0.956	0.772	1.183	0.679	Primary tumor origin	0.953	0.770	1.179	0.659
Tumor diameter	1.411	0.597	3.331	0.433	Tumor diameter	1.823	0.769	4.321	0.173
Lymph node involvement	4.438	2.360	8.344	<0.001	Lymph node involvement	10.101	4.975	20.510	<0.001
Tumor localization	1.032	0.827	1.287	0.782	Tumor localization	1.093	0.868	1.377	0.449
Metastatic site	1.653	0.935	2.921	0.084	Metastatic site	2.001	1.131	3.542	0.017
Postoperative complication	1.770	0.696	4.501	0.231	Postoperative complication	1.765	0.688	4.526	0.237
NLR	1.291	0.691	2.413	0.423	NLR	1.309	0.702	2.438	0.397
SII	1.240	0.579	2.654	0.580	SII	1.428	0.667	3.060	0.359
SIRI	2.180	1.208	3.936	0.010	SIRI	2.067	1.147	3.727	0.016
PIV	1.311	0.586	2.931	0.510	PIV	1.388	0.623	3.093	0.423
MAR	2.128	1.118	4.049	0.021	MAR	2.445	1.289	4.638	0.006

Abbreviations: ECOG PS, Eastern Cooperative Oncology Group performance status; RT, Radiotherapy; NLR, Neutrophil-to-lymphocyte ratio; SII, Systemic immune-inflammation index; SIRI, Systemic inflammation response index; PIV, Pan-immune-inflammation value; MAR, Monocyte-to-albumin ratio.

**Table 4:** Multivariate Cox Regression Analysis Identifying Independent Prognostic Factors for Overall and Progression-Free Survival.

Overall Survival	Exp(B)	95.0% CI for Exp(B)		*p*	Progression-Free Survival	Exp(B)	95.0% CI for Exp(B)		*p*
		Lower	Upper				Lower	Upper	
Lymph node involvement	4.526	2.340	8.751	<0.001	Lymph node involvement	14.124	6.089	32.761	<0.001
-	-	-	-	-	Metastatic site	2.019	1.095	3.720	0.024
SIRI	1.731	0.927	3.234	0.085	SIRI	1.893	1.021	3.509	0.043
MAR	2.002	1.022	3.920	0.043	MAR	2.189	1.114	4.303	0.023

Abbreviations: CI, Confidence interval; SIRI, Systemic inflammation response index; MAR, Monocyte-to-albumin ratio.

## Data Availability

The datasets generated and analyzed during the current study are available from the corresponding author upon reasonable request and with permission from the Thoracic Surgery Department of the University of Health Sciences Antalya Training and Research Hospital.

## Abbreviations

The following abbreviations are used in this manuscript:

AUC	Area under the curve
CT	Computed tomography
DFI	Disease-free interval
ECOG PS	Eastern Cooperative Oncology Group performance status
MAR	Monocyte-to-albumin ratio
NLR	Neutrophil-to-lymphocyte ratio
OS	Overall survival
PET-CT	Positron emission tomography–computed tomography
PFS	Progression-free survival
PIV	Pan-immune-inflammation value
ROC	Receiver operating characteristic
RT	Radiotherapy
SII	Systemic immune-inflammation index
SIRI	Systemic inflammation response index
TAM	Tumor-associated macrophage
VATS	Video-assisted thoracoscopic surgery
